# No evidence of XMRV in prostate cancer cohorts in the Midwestern United States

**DOI:** 10.1186/1742-4690-8-23

**Published:** 2011-03-29

**Authors:** Toshie Sakuma, Stéphane Hué, Karen A Squillace, Jason M Tonne, Patrick R Blackburn, Seiga Ohmine, Tayaramma Thatava, Greg J Towers, Yasuhiro Ikeda

**Affiliations:** 1 Department of Molecular Medicine, Mayo Clinic, Rochester, MN 55905 USA; 2 Department of Infection and Immunity, MRC Centre for Medical Molecular Virology, University College London, 46 Cleveland St, London W1T 4JF, UK

## Abstract

**Background:**

Xenotropic murine leukemia virus (MLV)-related virus (XMRV) was initially identified in prostate cancer (PCa) tissue, particularly in the prostatic stromal fibroblasts, of patients homozygous for the RNASEL R462Q mutation. A subsequent study reported XMRV antigens in malignant prostatic epithelium and association of XMRV infection with PCa, especially higher-grade tumors, independently of the RNASEL polymorphism. Further studies showed high prevalence of XMRV or related MLV sequences in chronic fatigue syndrome patients (CFS), while others found no, or low, prevalence of XMRV in a variety of diseases including PCa or CFS. Thus, the etiological link between XMRV and human disease remains elusive. To address the association between XMRV infection and PCa, we have tested prostate tissues and human sera for the presence of viral DNA, viral antigens and anti-XMRV antibodies.

**Results:**

Real-time PCR analysis of 110 PCa (Gleason scores >4) and 40 benign and normal prostate tissues identified six positive samples (5 PCa and 1 non-PCa). No statistical link was observed between the presence of proviral DNA and PCa, PCa grades, and the *RNASEL *R462Q mutation. The amplified viral sequences were distantly related to XMRV, but nearly identical to endogenous MLV sequences in mice. The PCR positive samples were also positive for mouse mitochondrial DNA by nested PCR, suggesting contamination of the samples with mouse DNA. Immuno-histochemistry (IHC) with an anti-XMRV antibody, but not an anti-MLV antibody that recognizes XMRV, sporadically identified antigen-positive cells in prostatic epithelium, irrespectively of the status of viral DNA detection. No serum (159 PCa and 201 age-matched controls) showed strong neutralization of XMRV infection at 1:10 dilution.

**Conclusion:**

The lack of XMRV sequences or strong anti-XMRV neutralizing antibodies indicates no or very low prevalence of XMRV in our cohorts. We conclude that real-time PCR- and IHC-positive samples were due to laboratory contamination and non-specific immune reactions, respectively.

## Background

Prostate cancer (PCa) is the most frequently diagnosed noncutaneous malignancy among men in industrialized countries [1]. Although early detection using tests for prostate-specific antigen and improved treatment have emerged as important interventions for decreasing PCa mortality, there is potential for improved prognosis through detection of genetic risk factors. Indeed, a positive family history is among the strongest epidemiological risk factors for PCa, and a number of genetic mutations have been implicated in PCa. For example, an R462Q polymorphism in the RNase L protein, which impairs the catalytic activity of an important effector of the innate antiviral response, has been implicated in up to 13% of unselected PCa cases [2].

Xenotropic murine leukemia virus (MLV)-related virus (XMRV) was first identified in PCa tissues, particularly those with the homozygous *RNASEL *R462Q mutation [3]. Genetic analysis identified XMRV as a xenotropic gammaretrovirus, closely related to those found in mice [4,5]. This suggested that XMRV represented a zoonotic transmission from mice to humans. When compared with exogenous and endogenous MLV sequences, XMRV appeared to have a unique, conserved 24 bp deletion in the *gag *leader region [3]. However, this deletion has recently been found in endogenous MLV proviruses in a variety of mice [6]. Initially, immuno-histochemistry (IHC) and FISH analyses suggested that only prostatic stromal fibroblasts were infected with XMRV [3]. Subsequently, Schlaberg, Singh and colleagues reported the expression of XMRV antigens in 23% of PCa and an association of XMRV infection with higher grade tumors [7]. Contrary to the initial study, Singh's study found viral antigen-positive cells primarily in malignant prostatic epithelium, independently of the *RNASEL *polymorphism [7]. It is notable that this study found many immuno-histochemistry-positive samples which did not have detectable XMRV DNA [7]. Another study found 11 (27.5%) of 40 PCa patients with XMRV neutralizing antibodies [8]. Importantly, there were correlations between serum positivity and nested PCR results, FISH, or the R462Q *RNASEL *mutation [8]. In sharp contrast, several recent reports found no or very low prevalence of XMRV (DNA, RNA or antibodies) in PCa samples [9-12].

If the role of XMRV in PCa is confirmed, detection and prevention of XMRV infection could provide a novel intervention strategy for early diagnosis and treatment of PCa. However, the conflicting epidemiological data have made it unclear whether XMRV plays a role in PCa and have questioned whether the virus is truly a human pathogen. In this study we have sought to address the association between XMRV infection and PCa, PCa grades and *RNASEL *R462Q polymorphism by testing prostate tissues for the presence of XMRV. In addition, to determine the correlation between PCa and seroprevalence of XMRV, serum samples from patients with PCa were compared with age-matched controls for detectable anti-XMRV antibodies. Our study found no XMRV sequences and no XMRV-neutralizing antibodies in 150 prostate tissues (110 PCa and 40 benign/normal) and serum samples (159 PCa and 201 age-matched controls), respectively, indicating no or very low prevalence of XMRV in our cohorts. We did detect MLV sequences in 6 samples, but these samples were also PCR positive for mouse mitochondrial DNA suggesting DNA contamination as a source of the MLV. We were therefore unable to confirm the links between XMRV infection with PCa, PCa grades or RNASEL mutation.

## Results

### Prevalence of XMRV proviral DNA in PCa

We have previously developed a real-time PCR assay for detection of XMRV *gag *sequences [13,14]. Tests using the XMRV infectious molecular clone plasmid, pcDNA3.1(-)/VP62, could detect a single copy of the XMRV genome in 1.0 μg of total cellular DNA (approximately 1.4 × 10^5 ^cells). The primers and the probe used in this assay were designed to detect most MLV-related sequences from mice. Using this sensitive real-time PCR assay, we screened DNA from 150 prostate tissues (110 PCa and 40 benign/normal controls). One out of 40 high grade PCa (Gleason score 8-10), 4 out of 70 intermediate grade PCa (Gleason score 5-7), and 1 out of 40 benign/normal prostate tissues (Gleason score <4) were repeatedly positive by this assay (Table 1). The viral DNA copy numbers ranged from 0.5 to 11 copies per 1.0 μg DNA (average of 4 reactions). As one diploid cell contains approximately 7.1 pg of DNA, we estimate that PCR-positive clinical samples had 0.5 to 11 copies of proviral DNA in 1.4 × 10^5 ^cells.

**Table 1:** Prevalence of XMRV and tumor grade

	**No.**^***a***^	**Positive**^***b***^
Low grade*^c^*	40	1
Gleason 5	2	0
Gleason 6	22	3
Gleason 7	46	1
Gleason 8	16	1
Gleason 9	23	0
Gleason 10	1	0

*^a ^*Total number of samples tested from each Gleason score.

*^b ^*Number of PCR positive samples from each Gleason score.

*^c ^*Low grade includes Gleason score 1 through 4.

To confirm the real-time PCR results, we screened the same DNA samples by nested PCR for XMRV/MLV *gag *sequences. In order to establish consistency and to minimize the risk of contamination during the procedure, three individuals independently performed the nested PCR experiments using independently aliquoted DNA samples. Four out of 6 real-time PCR positive samples (#15, 51, 52 and 112) were consistently positive by the nested PCR analysis, while the other two positive samples from intermediate grade PCa (#53 and 103) were shown to be nested PCR-positive twice in the first three attempts. Further analysis confirmed that these two samples were nested PCR-positive for viral DNA. The 144 real-time PCR-negative samples were also found to be negative by nested PCR.

### No statistical link between the presence of viral DNA and prostate cancer or higher tumor grade

We then sought a correlation between viral DNA detection and the presence of PCa. There was no statistical difference between the frequency of PCR positivity in PCa and in benign/normal controls (Table 2). We also examined a link between PCR positivity and tumor grade as measured by the Gleason score. Using total of 110 DNA samples from PCa, 4 out of 70 intermediate grade (Gleason score 5-7) and 1 out of 40 high grade (Gleason score 8-10) were positive by real time PCR (Table 3). These data were not statistically significant by chi-square (*x^2^*) as indicated in Table 3, suggesting no correlation between the prevalence of viral DNA and higher tumor grade in our samples.

**Table 2:** Statistical analysis of XMRV positivity in controls and PCa

	No.*^a^*	**Positive**^***b***^	***x***^***2c***^
Non-PCa*^d^*	40	1	0
PCa*^e^*	110	5	0.319

*^a ^*Total number of samples tested from each Gleason score.

*^b ^*Number of PCR positive samples from each Gleason score.

*^c ^*Statistical results from chi-square (*x^2^*) tests.

*^d ^*Samples from benign/normal cancer patients.

*^e ^*Samples from prostate cancer patients.

**Table 3:** Statistical analysis of XMRV prevalence and tumor grade

	**No.**^***a***^	**Positive**^***b***^	***x***^***2c***^
Low grade	40	1	0
Intermediate	70	4	0.606
High grade	40	1	0

*^a ^*Total number of samples tested from each Gleason score.

*^b ^*Number of PCR positive samples from each Gleason score.

*^c ^*Statistical results from chi-square (*x^2^*) tests.

### No correlation between viral DNA detection and RNASEL R462Q mutation

In order to consider the association between *RNASEL *mutation and viral infection, we amplified part of the *RNASEL *gene by PCR and determined the status of the R462Q *RNASEL *polymorphism. Of 150 prostate tissues, 20 cases were found to be homozygous for RNASEL R462Q (Table 4). However, these samples were all negative for viral DNA by real-time PCR. Thus there was no linkage between viral DNA detection and *RNASEL *R462Q in our clinical samples (Table 5).

**Table 4:** *RNASEL *genotyping and tumor grade

	**Normal/benign**^***a***^	**Intermediate**^***b***^	**High**^***c***^	**Total**^***d***^
*RNASEL *RR	11	28	15	54
*RNASEL *RQ	21	36	19	76
*RNASEL *QQ	8	6	6	20

*^a ^*Samples with Gleason score 1 through 4.

*^b ^*Samples with intermediate Gleason score.

*^c ^*Samples with high Gleason score.

*^d ^*Total numbers of each *RNASEL *genotypes.

**Table 5:** Statistical analysis of XMRV prevalence and *RNASEL *genotyping

	XMRV+*^a^*	XMRV-*^b^*	*x^2c^*
*RNASEL *RR+RQ	6	124	0
*RNASEL *QQ	0	20	0.962

*^a ^*XMRV positive samples from real time PCR.

*^b ^*XMRV negative samples from real time PCR.

*^c ^*Statistical results from chi-square (*x^2^*) tests.

### Phylogenetic analyses of MLV-like sequences in prostate tissue DNA

**Figure 1:**
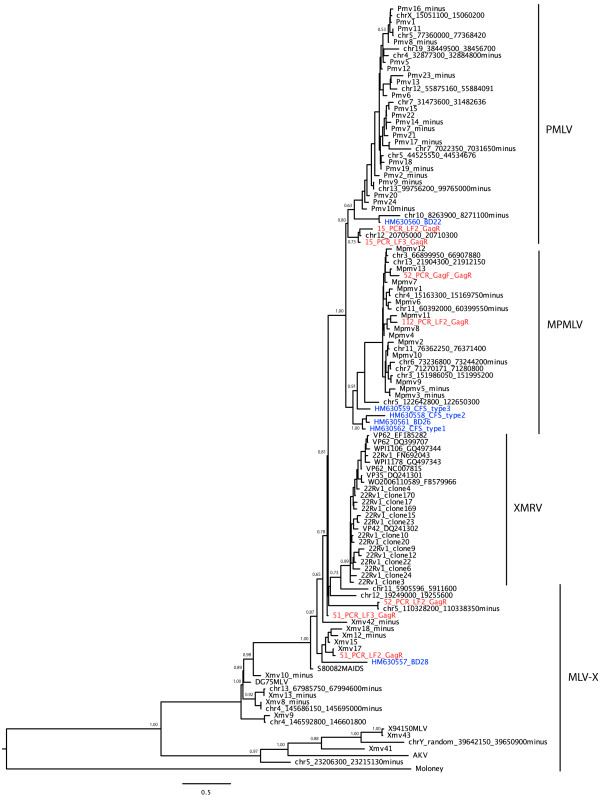
**Bayesian maximum clade credibility phylogeny of endogenous murine MLV sequences, 22Rv1 cell line and patient derived MLV *gag *gene sequences**. Sequences derived from PCa samples in this study are colored red. Sequences from [16] are colored blue. The tree is rooted against the Moloney MLV sequence. Bayesian posterior probabilities above 0.50 are indicated on the corresponding branches. The scale bar represents the number of nucleotide substitutions per site.

XMRV has been PCR amplified from prostate cancer samples in a number of studies [3,8,12] as well as in blood samples from patients with chronic fatigue syndrome (CFS) [15]. Furthermore, a recent study reported a high frequency of MLV that was distinct from XMRV by PCR in patients with chronic fatigue syndrome [16]. To examine the viral sequences identified in our PCa samples, we cloned the PCR-amplified DNA bands from four viral DNA-positive patient samples (#15 [GenBank no. JF288880, JF288881], #51 [GenBank no. JF288878, JF288879], #52 [GenBank no. JF288882, JF288883] and #112 [GenBank no. JF288884]) and determined their nucleotide sequences. We were able to identify two independent sequences from each of patients #15, #51 and #52 and a single sequence from patient #112. To compare these sequences to XMRV and to previously published MLV sequences from mice and patient samples, we reconstructed Bayesian phylogenies (Figure 1). None of the *gag *gene sequences amplified from our clinical samples belonged to the clade formed by previously reported XMRV sequences; instead, they clustered with known polytropic murine leukemia virus (PMLV), modified polytropic murine leukemia virus (MPMLV) or xenotropic murine leukemia virus (MLV-X) endogenous sequences of mice (Figure 1). Importantly, one of the patients (#52) appeared to be infected with two independent MLVs, one from the modified polytropic MLV clade and one from the xenotropic MLV clade. A similar result was seen when a maximum likelihood phylogeny was constructed using the software RAxML [17] (not shown). In each case, BLAST analysis of the amplified sequences identified at least one endogenous MLV sequence in the mouse genome with very high (>99%) similarity (Table 6). Two of the five fragments were identical to known endogenous proviruses and the other three were greater than 99% similar. These proviruses exist in multiple locations within the mouse genome.

**Table 6:** Comparison of MLV sequences amplified from patient samples with mouse genomic sequences

Sequence (GenBank no.)	Length (nt)	Closest relative	**GenBank no**.	Similarity	Nucleotide difference
51_PCR_LF2_GagR (JF288878)	608	Mus musculus BAC clone RP23-457E5	AC121813	100%	0/608
51_PCR_LF3_GagR (JF288879)	250	Mus musculus chrom 7, clone RP24-220N8	AC167466	99%	1/250
15_PCR_LF2_GagR (JF288880)	608	Mus musculus BAC clone RP23-152O2	AC163634	100%	0/608
15_PCR_LF3_GagR (JF288881)	271	Mus musculus BAC clone RP23-152O2	AC163634	>99%	1/271
52_PCR_GagF_GagR (JF288882)	525	Mouse DNA sequence, clone CH29-187G15	CU407131	100%	0/525
52_PCR_LF2_GagR (JF288883)	540	Mus musculus chrom 5, clone RP23-280N22	AC123679	>99%	1/540
112_PCR_LF2_GagR (JF288884)	691	Mouse DNA sequence, CH29-187G15	CU407131	>99%	6/691

NB: Gaps are treated as mismatches

Because the sequences we amplified were similar to the MLV sequences detected in CFS patients [16], we also analyzed the sequences reported in that study. The sequences amplified from CFS patients also fell into both polytropic and modified polytropic clades of endogenous MLVs (Figure 1). They were also very similar (98-100%) to known endogenous MLV proviruses in mice (Table 7). In fact, the differences between the amplified sequences and the endogenous sequences are consistent with known error rates of Taq polymerase or could also be explained by polymorphisms between mice [18-20].

**Table 7:** Comparison of MLV sequences amplified from patient samples [16] with mouse genomic sequences

Sequence	Length (nt)	Closest relative	**GenBank no**.	Similarity	Nucleotide difference
HM630557	319	Mouse endogenous retrovirus	M26006	99%	4/310
HM630558	698	Mus musculus BAC clone RP23-115O21	AC163617	99%	7/698
HM630559	698	Mouse DNA sequence from clone RP23-131N18	AL772224	99%	1/697
HM630560	697	Mouse endogenous retrovirus	M26005	99%	3/698
HM630561	339	Mouse DNA sequence, clone CH29-187G15	CU407131	99%	6/339
HM630562	698	Mus musculus BAC clone RP23-115O21	AC163617	99%	5/697

Patient amplified MLV sequences were used as a BLAST query to identify their closest relative. The accession numbers of the mouse genomic sequences identified are shown as are the number of nucleotide differences between the patient amplified sequence and their nearest relatives in the mouse genome.

**Figure 2:**
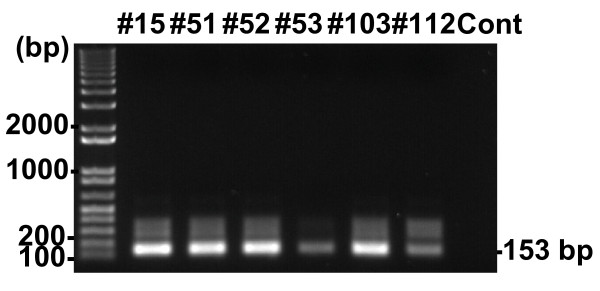
**PCR for mouse mitochondrial DNA**. qPCR positive samples (#15, 51, 52, 53, 103, 112) were PCR amplified for mouse mitochondrial DNA. Positive samples yielded PCR products at 153 bp [16]. Water was used as a control.

The similarity between the patient amplified sequences and known endogenous MLV provirus sequence in mice suggests that the MLVs may have been amplified from samples that had been inadvertently contaminated with mouse DNA. To examine this possibility further in our samples we tested each positive PCa sample for the presence of mouse mitochondrial DNA by PCR. Strikingly, all of the clinical samples that were positive for MLV were also positive for mouse mitochondrial DNA (Figure 2). When the amplified DNA fragments were cloned and sequenced, they were 100% identical to *Mus musculus *cytochrome b gene sequence. Thus, the MLV sequences detected by sensitive PCR methods in patient samples likely originated from contaminating mouse DNA encoding endogenous MLV proviruses.

### Detection of XMRV antigens in PCa tissues

**Figure 3:**
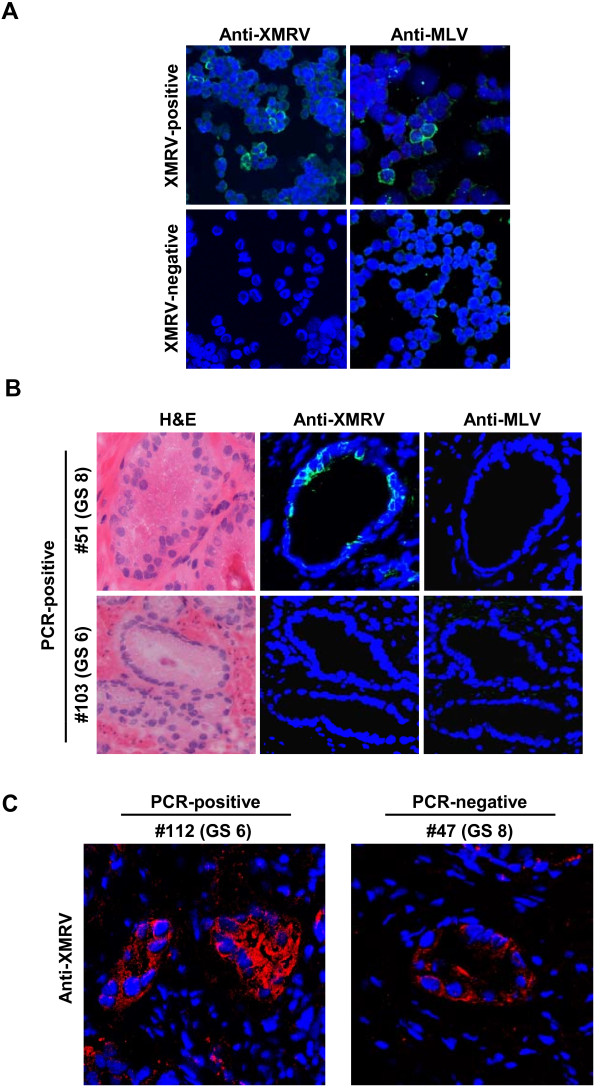
**Detection of XMRV in prostate cancer tissues**. (A) Specificity of anti-XMRV antiserum and anti-MLV antibody. 293T cells transfected with XMRV infectious plasmid (pcDNA3.1(-)/VP62) were stained with either rabbit anti-XMRV or goat anti-MLV. No positive staining was observed in control uninfected 293T cells. (B) Serial tissue sections from qPCR positive samples, including #51 (Gleason score (GS) 8) and #103 (GS 6) were immunostained with either anti-XMRV or anti-MLV antibody. H&E staining from each sample is also shown. (C) Serial tissue sections from qPCR positive (#112, GS 6) and negative (#47, GS 8) samples were immunostained with anti-MLV antibody, followed by TexasRed-conjugated donkey anti-rabbit antibody (Jackson ImmunoResearch Laboratories, Inc., 1:200).

Previous IHC studies found XMRV antigen-positive cells in prostatic stromal fibroblasts [3] or in malignant prostatic epithelium [7]. Importantly, Schlaberg, Singh and colleagues also showed frequent detection of viral antigen-positive cells in PCR-negative tissues [7]. In order to seek viral antigen-positive cells in our clinical samples, we prepared prostate tissue sections and performed IHC analysis. We used the rabbit anti-XMRV antibody, which was used in the previous study by Schlaberg et al. [7]. We also used a goat anti-MLV p30/gp70 antibody, which can detect XMRV precursor Gag, CA, and Env proteins in XMRV transfected cells [13,14]. Both antisera showed clear and reproducible staining of 293T cells transfected with the infectious XMRV clone VP62 (Figure 3A) or XMRV-producing 22Rv1 cells (data not shown). No specific staining was seen when uninfected control 293T cells were stained with these antisera (Figure 3A). We prepared tissue sections of four PCR-positive tissues (Gleason scores 6 and 8) as well as two PCR negative tissues (Gleason scores 6 and 8, real-time/nested PCR-double negative), and analyzed them with the two antisera. The anti-XMRV antibody sporadically detected antigen-positive cells, exclusively in prostatic epithelium, in the sections of tissues (Figure 3B, upper middle panel with FITC). Similar results were observed with a different secondary antibody conjugated with Texas Red (Figure 3C). In contrast, no signal was detected with the anti-MLV p30/gp70 in any of the tissue sections (Figure 3B). Importantly, the anti-MLV serum did not stain the cells, which were shown to be IHC-positive by the anti-XMRV serum, in the serial sections of the same tissue (Figure 3B, upper panels). It was also notable that the anti-XMRV serum found antigen-positive cells in PCR-negative tissue sections (Figure 3C), suggesting that this serum also recognizes a non-viral protein. Similar results were recently reported by Switzer et al [21]. Considering the data obtained using the anti-MLV serum, we conclude that we cannot detect XMRV in prostate cancer tissues and that the antibody described by Schlaberg, Singh and colleagues recognizes non-viral proteins in addition to XMRV.

### Absence of XMRV antibodies in patients with PCa and age-matched controls

**Figure 4:**
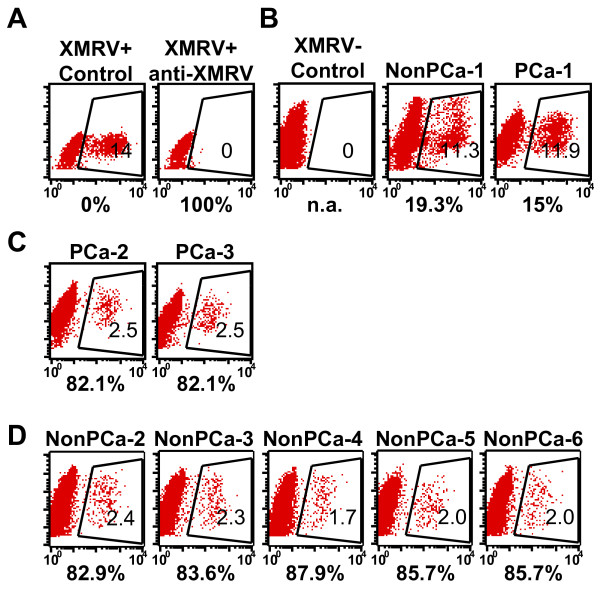
**Neutralization activity of patient sera**. (A) XMRV infected 293T cells (XMRV+ control) and XMRV-infected and treated with anti-XMRV sera at a dilution of 1:10 [14] are shown. (B) Data from non-XMRV infected 293T cells is shown as a control. Patients samples which did not show positive neutralization reaction (Patients with non-prostate cancer (NonPCa)-1, Patients with prostate cancer (PCa)-1) are shown. (C) Two samples that showed positive reaction from patients with prostate cancer (PCa-2 and -3) are shown. (D) Six samples that showed positive neutralization reaction from patients with non-prostate cancer (NonPCa-2 to -6) are shown. 1:10 dilution of sera were applied for all the experiments. Percent GFP positive and percent neutralization are indicated within the gated areas and below the flow data, respectively. The percent neutralization was calculated as the reciprocal of infectivity, with a maximum infectivity being determined by incubation of the virus with an uninfected mouse serum. n.a., not applicable.

Serological testing was performed with a recently developed XMRV neutralizing assay which measures viral neutralizing activity using a GFP-encoding XMRV and flow cytometry [14]. Positive seroreactivity was defined as 100% block of XMRV-GFP transduction with a 10-fold diluted serum sample. We randomly sampled 159 PCa cases out of 933 patients who are consenting, age 50-70 and have a clinical Gleason Score of 6 or 7 (most common) in the Mayo Clinic Prostate SPORE Biospecimen files. 201 sera from age-matched patients without PCa or any known urological disorders were included as non-PCa controls. As positive controls, we used anti-XMRV sera from XMRV-infected wild mice, *Mus pahari *[14]. Sera from XMRV-infected mice diluted 10-fold completely blocked XMRV-GFP infection (Figure 4A). In contrast, none of the clinical samples showed strong anti-XMRV activity at 10-fold dilution (Figure 4B). Two out of 159 PCa (Figure 4C) and five out of 201 non-PCa (Figure 4D) sera marginally reduced the XMRV infectivity (over 80% block of XMRV infectivity at a 10-fold dilution). However, by Western blot probing cell lysates from XMRV-infected and uninfected cells, these patients' sera failed to detect XMRV Env, Gag or p30 Capsid (data not shown). To rule out the possibility that these patients' sera cannot detect denatured XMRV proteins by Western blotting, we also performed the indirect immunofluorescent assay using HeLa cells (control) and XMRV-infected HeLa cells as antigens. None of the sera could detect XMRV antigens in HeLa cells at 50- and 200-fold dilutions (data not shown). We, therefore, conclude that XMRV antibodies are absent from our patient population.

## Discussion

In this study, we have examined the prevalence of XMRV in patients with or without PCa at Mayo Clinic. We were unable to find XMRV sequences or anti-XMRV antibodies in our patients, most of whom are from the mid-west area of the USA, indicating that there is no or very low prevalence of XMRV in this region. Moreover, we were unable to confirm the correlation between XMRV infection and PCa, higher tumor grade or *RNASEL *R462Q mutation.

A high prevalence of XMRV has been reported in patients with PCa and chronic fatigue syndrome (CFS) in the USA [3,7,8], but similar studies in Europe have failed to detect XMRV [10-12]. It has been suggested that geographical differences might explain this striking variation in XMRV prevalence [11] but our results, as well as recent US studies that also find no evidence for XMRV [9,21], appear to rule this explanation out. In this regard, it is notable that previous studies to identify XMRV in patients with PCa or chronic fatigue syndrome have relied on very sensitive PCR detection methods. Because of the high similarity between patient associated XMRV/MLV and endogenous MLV sequences and the striking discordance between studies, it has been suggested that PCR-positive results might be attributed to unintentional detection of contaminating mouse DNA in human specimens [6,22-24]. It is notable that Lo et al. [16] detected polytropic and modified polytropic MLV sequences, but not XMRV, in blood samples from chronic fatigue patients (Figure 1). These authors were unable to identify the samples as contaminated using mouse mitochondrial PCR. In our study, real-time PCR and nested PCR identified 6 of 150 samples as positive for MLV. However, the amplified sequences were closely related to known endogenous MLV proviruses, rather than XMRV. In fact one patient sample (#52) contained two independent MLV sequences. This might be interpreted as evidence for evolution of the virus in the patient but closer analysis reveals that one of the sequences is identical to a known endogenous modified polytropic sequence whilst the other is a single nucleotide different from a known mouse endogenous xenotropic MLV. This, therefore, suggests either infection of this patient with two independent MLVs or PCR contamination with mouse DNA as a source. As all of the MLV PCR-positive samples contained detectable levels of mouse mitochondrial DNA, we conclude that the amplified sequences originated from mouse DNA that somehow contaminated the study samples.

In order to confirm that the viral sequences were amplified from endogenous MLV in mouse genomic DNA, but not replicating MLV in human tissue, we attempted to determine viral integration sites. We first used the protocol described by Kim *et al. *[25] but failed to amplify DNA sequences containing the partial XMRV LTR. We then designed universal primers to recognize LTRs from XMRV and endogenous and exogenous MLVs [26], as well as a series of primers specific for the viral sequences identified in our clinical samples. Unfortunately, we were not successful, likely due to low viral copy numbers in the clinical samples. Very recently, Robinson et al. [23] and Oakes et al. [22] reported similar observations; all XMRV PCR-positive specimens contained detectable levels of mouse mitochondrial or endogenous retroelements (IAPs). Together with our data, these findings highlight the difficulty of avoiding DNA contamination in clinical samples and the risk of testing contaminated samples as XMRV-positive by sensitive PCR detection assays. As a possible source of contamination, Sato et al. [24] demonstrated that a commercially available hot-start PCR enzyme contained mouse DNA. We used several enzymes and obtained similar results. Thus, it is unlikely that the contaminating mouse genome originated from a PCR kit. Since we could amplify the viral sequences from multiple aliquoted DNA samples, they appeared to be contaminated before or during the DNA isolation step, most likely during tissue sectioning on a microtome.

XMRV antigen-positive cells have been detected in prostatic stromal fibroblasts [3] or in malignant prostatic epithelium [7]. Our IHC study using two different antisera showed conflicting results. The goat anti-MLV antibody found no viral antigens in clinical samples, while the rabbit anti-XMRV antibody used in the study by Schlaberg, Singh and colleagues [7] detected antigen-positive cells in prostatic epithelium. Strikingly, the goat anti-MLV serum did not stain the cells, which were IHC-positive by the anti-XMRV rabbit serum, in serial sections of the same tissue. The rabbit antiserum also found antigen-positive cells in PCR-negative sections, confirming the observations of Schlaberg and colleagues who reported frequent detection of IHC-positive samples in PCR-negative tissues [7]. Importantly, both the rabbit and goat antibodies detected XMRV in experimentally infected cells with high sensitivity (Figure 3). Together, these observations strongly suggest that the rabbit antiserum is detecting a non-viral antigen sporadically expressed by tumor cells in the tissue section. We conclude that our PCa samples do not have XMRV antigen-expressing cells that are detectable by IHC.

We recently reported that *Mus pahari *mice elicit potent XMRV-specific humoral immune response upon XMRV infection [14]. At a serum dilution of 1:640, antisera from infected animals almost completely blocked XMRV infection [14]. Similarly, an animal study using XMRV-infected rhesus macaques and sensitive ELISA detection assays showed that infected animals rapidly develop antibodies against XMRV proteins, including gp70 (Env), p15E (transmembrane), and p30 (CA) [27]. These results indicate that XMRV is strongly immunogenic in these animals. In contrast, we were unable to detect strong XMRV-specific neutralizing antibodies in our 360 patients, age 50-70, with or without PCa. This observation further suggests a lack of XMRV in our cohorts. It is possible, although less likely, that XMRV is not immunogenic in humans or that XMRV-specific immune response might have disappeared in these relatively elderly patients.

## Conclusion

In our study population of patients with or without PCa from the USA, we found no evidence of infection with XMRV using PCR, IHC and serological tests. Our negative results are in accordance with previous studies using sensitive PCR, ELISA and Western blot assays, which failed to detect PCR or seropositive samples in a large number of blood donors, HTLV- and HIV-infected, or patients with or without CFS [9-12,21,27-31]. Our results indicate the possible false-positive detection of XMRV/MLV-related sequences or antigen-positive cells through laboratory contamination or non-specific immune reaction respectively, and underscore the need for careful validation of previous and future studies.

## Materials and methods

### Prostate tissues and plasma samples from patients

Prostate tissues and plasma samples were obtained from Mayo Clinic Biospecimen Core with an approval from the Institutional Review Boards. Frozen sections of prostate cancer tissues (10 μm) were identified as 1 through 150 in duplicates. These samples included 40 normal/low grade Gleason score, 70 intermediate (Gleason score 5-7), and 40 high grade (Gleason score 8-10) with men aged between 50-70 years old. For plasma analysis, total of 360 plasma samples from 50-70 year old male patients including 159 prostate patients (Gleason score 5-7) and 201 patients with no prostate cancer or urological disorders were used in this study.

### TaqMan qPCR

Total cellular DNA was extracted by PureLink Genomic DNA Mini Kit according to the manufacturer's protocol (Invitrogen). All samples were eluted in 50 μl of elution buffer, and the concentration and quality of the DNA were determined by a NanoDrop Spectrophotometer. For the real-time PCR assay, TaqMan Universal PCR Master Mix (Roche) was used along with 2 μl of each sample. Primers were used at a range of 230 nM to 300 nM final concentration. TaqMan probe #51 from Roche Universal Probe Library was used for XMRV-*gag *at 100 nM final concentration. A standard curve was created by using serially diluted XMRV plasmid (pcDNA3.1(-)/VP62). The assay was analyzed by the ABI 7300 Real-Time PCR System using the default thermal cycling conditions for the two-step RT-PCR method and FAM reporter [13].

### Genotyping

*RNASEL *genotype was determined by nested PCR amplification using outer primers 5'-CTGGGGTTCTATGAGAAGCAAG-3' and 5'-TGAGCTTTCAGATCCTCAAATG-3', and inner primers 5'-GAGAGAACAGTCACTTGGTGAC-3' and 5'-CAGCCCACTTGATGCTCTTATC-3' with *pfx *polymerase (Invitrogen). Final PCR products were purified with QIAquick PCR Purification Kit (Qiagen) before sequence analysis.

### Neutralization assay

The neutralization assay was carried out using GFP-encoding XMRV as described previously [13,14]. Briefly, 293T cells were transfected with pcDNA3.1(-)/VP62 and a GFP-encoding retroviral vector using FuGene 6 (Roche). Serum samples were heat inactivated at 56°C for 30 min. A mixture of plasma samples and 2.5 × 10^4 ^infectious units of GFP-carrying XMRV were incubated at 37°C for 30 min before infecting 293T cells (5 × 10^4^). Three days post-infection, cells were resuspended, fixed with 4% paraformaldehyde and analyzed by flow cytometry (BD FACScan). The percentages of GFP-positive cells were measured using CellQuestPro software [14].

### Western blot analysis

For Western blot analysis of XMRV proteins, cell lysates of prostate cancer (PC-3) cell line (ATCC) and PC-3 infected with XMRV were harvested in 1.0 ml of RIPA lysis buffer. Cell debris was removed by centrifugation, and the supernatant was diluted with Laemmli sample buffer containing β-mercaptoethanol. After heat-denaturation at 95°C for 5 min, 10 μl of proteins were subjected to SDS-PAGE with a 4-15% gradient gel (Bio-Rad), and transferred to a polyvinylidene diflouride membrane at 0.7 mA/cm^2 ^for 40 min. Membranes were blocked in 5% milk/PBS, then stained with patient's plasma samples diluted to 1:250, followed by anti-human IgG (1:1000, Jackson ImmunoResearch Laboratories, Inc.).

### Immuno-histochemistry

Immunohistochemistry was performed on tissue samples from patients with or without prostate cancer. Sections were fixed with 4% paraformaldehyde for 20 min and treated with 0.3% Triton X100 for 15 min at room temperature. They were then blocked with 5% FBS/PBS for 30 min and immunostained with rabbit-anti XMRV (kindly provided by Dr. Ila Singh, University of Utah) or goat-anti p30/gp70 (NCI HD625 CAT No. 04-0109, LOT No. 81S000262, Quality Biotech, kindly provided by Dr. Yasuhiro Takeuchi, UCL) at a dilution of 1:500 for 4 h at room temperature. FITC-conjugated anti-rabbit IgG (1:500; Amersham) or DyLight 488-conjugated anti-goat IgG (1:500; Jackson ImmunoResearch Lab) were applied for 2 h at room temperature. Nuclei were then counter-stained with 4'-6-Diamidino-2-phenylindole (DAPI), and analyzed by confocal microscopy (Zeiss).

### Nested PCR and sequence analysis of proviral DNA

Sequence analysis was performed as previously described [14]. Briefly, DNA was extracted by PureLink Genomic DNA Mini Kit (Invitrogen). Nested-PCR was performed for XMRV *gag *(primers for outer *gag*: 5'-ACGAGTTCGTATTCCCGGCCGCA-3' and 5'-CCGCCTCTTCTTCATTGTTC-3', primers for inner *gag*: 5'-GCCCATTCTGTATCAGTTAA-3' and 5'-AGAGGGTAAGGGCAGGGTAA-3') with platinum *Taq *polymerase (Cat. no. 10966-034, Invitrogen). The resulting PCR products from a total of 4 patient samples (#15, #51, #52 and #112) were cloned into the TOPO vector (Invitrogen). Sequences from the two patient samples #53 and #103 were not analyzed. From patients #15, 51, 52, 112, we sequenced 2, 2, 4, 1 clones, and got 2, 2, 2, 1 different sequences, respectively. They were analyzed by DNADynamo (BlueTractorSoftware).

### Phylogenetic analyses

Seven unique *gag *gene sequences (255 to 528 nt), amplified from our clinical samples (GenBank no. JF288878, JF288879, JF288880, JF288881, JF288882, JF288883, and JF288884), were manually aligned with previously described murine leukemia virus *gag *gene sequences (n = 79), 22Rv1 cell line derived *gag *sequences (1605 nt; n = 15), XMRV *gag *sequences apparently amplified from prostate cancer and CFS samples (n = 7) [6], as well as 6 MLV virus *gag *sequences isolated from chronic fatigue syndrome samples [16]. Bayesian phylogenies were reconstructed as previously described [6]. The Markov chain Monte Carlo search was set to 10,000,000 iterations, with trees sampled every 1000th generation, and with a 20% burn in. The phylogeny of the aforementioned sequences was also reconstructed by maximum likelihood (ML) inference under the general time reversible model of nucleotide substitution, with gamma-distributed rate heterogeneity and proportion of invariable sites, using the program RAxML (data not shown) [32]. The ML topology was assessed by neighbor joining bootstrapping with 1000 replicates using the program PAUP*.

### A semi-nested mouse-specific mtDNA PCR

We used a PCR assay for mouse mitochondrial DNA reported to be able to detect 2.5 fg of mouse DNA in the presence of 35 ng human background DNA [16]. Using this assay, we tested whether our samples were contaminated with mouse DNA. DNA from PCR positive samples were PCR amplified with KOD Hot Start DNA Polymerase following the manufactures instruction (Novagen) as described [16]. The resulting PCR fragments were further cloned into the TOPO vector and the sequences were confirmed to be identical to the mouse cytochrome b gene by DNA BLAST.

## Competing interests

The authors declare that they have no competing interests.

## Authors' contributions

TS, SH, KAS, JMT, and PRB performed experiments. TS, SH, GT and YI designed the experiments, analyzed the data and wrote the paper. All authors read and approved the final manuscript.
